# Sex Differences and Menstrual Cycle Phase-Dependent Modulation of Craving for Cigarette: An fMRI Pilot Study

**DOI:** 10.1155/2014/723632

**Published:** 2014-11-13

**Authors:** Adrianna Mendrek, Laurence Dinh-Williams, Josiane Bourque, Stéphane Potvin

**Affiliations:** ^1^Centre de Recherche de l'Institut Universitaire en Santé Mentale de Montréal, 7331 rue Hochelaga, Montreal, QC, Canada H1N 3V2; ^2^Department of Psychiatry, Faculty of Medicine, University of Montreal, CP 6128, Succursale Centre-Ville, Montréal, QC, Canada H3C 3J7; ^3^Department of Psychology, Bishop's University, 2600 rue College, Sherbrooke, QC, Canada J1M 1Z7

## Abstract

While overall more men than women smoke cigarettes, women and girls take less time to become dependent after initial use and have more difficulties quitting the habit. One of the factors contributing to these differences may be that women crave cigarettes more than men and that their desire to smoke is influenced by hormonal fluctuations across the menstrual cycle. Therefore, the purpose of the present study was twofold: (a) to examine potential sex/gender differences in functional neuroanatomy of craving and to (b) delineate neural correlates of cigarette cravings in women across their menstrual cycle. Fifteen tobacco-smoking men and 19 women underwent a functional MRI during presentation of neutral and smoking-related images, known to elicit craving. Women were tested twice: once during early follicular phase and once during midluteal phase of their menstrual cycle. The analysis did not reveal any significant sex differences in the cerebral activations associated with craving. Nevertheless, the pattern of activations in women varied across their menstrual cycle with significant activations in parts of the frontal, temporal, and parietal lobe, during follicular phase, and only limited activations in the right hippocampus during the luteal phase.

## 1. Introduction 

Although the number of smokers is gradually decreasing in the Western world, the decline has been less pronounced in women than in men [1, 2]. In fact, international and local epidemiological reports from the World Health Organization and the Quebec (Canada) Statistics Institute suggest that cigarette smoking is on the rise in young women and teen girls [3, 4]. Among adults still more men than women smoke, but women take less time to become dependent after initial use, report shorter and less frequent abstinence periods, smoke for longer periods of time in their lives, and have more difficulties quitting the habit, than men [5–7].

The reasons for these sex/gender differences remain unclear, but one of the contributing factors may be that women crave cigarettes with greater intensity than men do. For example, Field and Duka [8] showed that cigarette craving was increased in the presence of smoking cues in women but not in men.

Regardless of sex differences, several functional imaging studies have been performed on cigarette cue reactivity. Recently, a meta-analysis of 11 functional imaging studies has shown that cigarette cues evoke activations in the anterior extended visual system, precuneus, anterior and posterior cingulate gyri, medial and dorsal prefrontal cortex, and insula and dorsal striatum [9]. When examining the influence of sex differences, Knott and colleagues [10] employed self-reports and electroencephalography (EEG) to examine the neural basis of cue-elicited cigarette craving and found that frontal EEG alpha asymmetry (evident with cigarette-cue exposure) was particularly pronounced in female smokers. In a rare fMRI study by McClernon et al. [11], women exhibited larger cue reactivity in the right putamen, bilateral cuneus, and left middle temporal gyrus, while men had greater responses in the left hippocampus and left orbitofrontal cortex.

These sex/gender differences have been attributed to the psychosocial as well as biological factors including the influence of ovarian hormones in females. Indeed, recent reports in human smokers reveal higher levels of craving and relapse in women who quit smoking during the early follicular phase (associated with relatively low estradiol and progesterone levels) than in those who quit during the luteal phase (associated with higher estradiol and progesterone) [12, 13].

Thus, the purpose of the present study was twofold: to examine potential sex differences in functional neuroanatomy underlying craving and to delineate neural correlates of cigarette cravings in women across their menstrual cycle (follicular versus luteal phase).

## 2. Methods

### 2.1. Participants

Thirty-four healthy smokers (19 women and 15 men) were recruited through the research center and affiliated hospital, as well as through Internet advertisements. Participants were chronic smokers not currently seeking treatment and right-handed (except for 1 ambidextrous and 1 left-handed), with no concomitant neurological or psychiatric disorders and no contraindication for magnetic resonance imaging (MRI).

Nicotine dependence severity was assessed using the Fagerström Test for Nicotine Dependence (FTND) [14] and cigarette craving with the French Tobacco Craving Questionnaire (FTCQ-12) [15] prior to the functional magnetic resonance imaging (fMRI) scanning procedure. We administered the Beck Depression Inventory (BDI) [16] and the State-Trait Anxiety Inventory (STAI) [17] as measures of depression and anxiety symptoms, respectively.

Men were tested once, while 13 women were tested twice in a counterbalanced manner (9 did follicular-luteal testing; 4 did luteal-follicular testing): once between day 3 and day 8 of their menstrual cycle (early follicular phase: mean = 5.4 days (SD = 2.5)) and once between day 16 and day 26 of the cycle (depending on the length of the cycle; midluteal phase: mean = 21.1 days (SD = 2.8)). Women were tested approximately 2 weeks apart (mean = 18.8 days (SD = 10.8)). Only women with a regular menstrual cycle (ranging from 24 to 32 days) were included in the study.

The menstrual phase and menstrual history were initially determined from the Menstrual Cycle Questionnaire and then verified through the hormonal assays due to the high rate of unreliability in menstrual cycle self-reports. A blood sample of 10 mL was taken approximately 30 minutes prior to each scanning session to evaluate the levels of estradiol, progesterone, and testosterone, using the automated chemiluminescence assay system [18] (SYNCHRON LXi 725, Beckman Coulter, USA) (for details see [18]). We calculated the estrogen to progesterone ratio (E : P ratio) to better differentiate between the follicular and luteal phases of the cycle [19]. Progesterone is significantly higher during the luteal phase of the cycle (peak at midluteal), while estrogen is the main hormone controlling the follicular phase. Thus, out of the two blood samples taken in the span of two weeks, the one with the significantly higher E : P ratio was indicative of the follicular phase while the blood sample with the lower E : P ratio was indicative of the luteal phase.

In agreement with the* Declaration of Helsinki*, written informed consent was obtained from each participant prior to the testing sessions. The study was approved by the Ethics Committee of the* Réseau de Neuroimagerie du Québec*.

### 2.2. fMRI Procedure

Thirty to 40 minutes prior to each fMRI scanning session, participants were invited to smoke a cigarette to minimize withdrawal effects and standardize the period of nonsmoking, while in the scanner, following the anatomical acquisition, participants passively viewed an alternating sequence of appetitive smoking-related images from the International Smoking Image Series (ISIS) [20] and neutral pictures taken from the International Affective Picture System (IAPS) [21]. Neutral IAPS pictures were matched with the smoking-related images (ISIS) for visual complexity, color, and number of faces and body parts.

During the scanning session, participants were instructed to press a button when a picture appeared in order to monitor their level of attention. The task consisted of an alternating sequence of 5 experimental (appetitive smoking-related images) and 5 control condition blocks (neutral pictures) with 10 periods of rest separating the blocks from one another. The rest period consisted of a 15-second blank screen with a fixation cross. Each block lasted for 25 seconds and consisted of 5 pictures, presented for 4 seconds each. There was an interstimulus interval (blank screen) of an average of 1 second (ranging from 0.5 to 1.5) presented before each picture. Within a block, images were randomly presented. Participants viewed a total of 25 appetitive as well as 25 neutral pictures.

At the end of the fMRI session, participants were represented with the smoking-related and neutral images and were asked to rate them on a scale from 0 (images elicit no desire to smoke a cigarette) to 100 (images elicit the strongest desire to smoke ever experienced).

### 2.3. fMRI Data Acquisition

We recorded blood oxygenation level dependent signals using a single-shot, gradient-recalled echo-planar imaging sequence (repetition time = 3000 ms, echo time = 30 ms, flip angle = 90°, matrix size = 64 × 64 voxels, and voxels size = 3.5 × 3.5 × 3.5 mm^3^) on a Siemens TRIO MRI system at 3.0 Tesla at the* Functional Neuroimaging Unit at the University of Montreal Geriatric Institute*. We then registered the functional volumes to individual high-resolution coplanar anatomical images taken during the same scanning session.

### 2.4. fMRI Data Analysis

We analyzed fMRI data using statistical parametric mapping software (SPM5: Wellcome Department of Cognitive Neurology, London, UK) according to the methods outlined by Friston et al. [22]. The functional images were realigned to the mean volume of the run to correct for artifacts due to minor head movements and high-pass filtered, spatially normalized into the standardized brain template and spatially smoothed with a three-dimensional isotropic Gaussian kernel (8 mm FWHM) to improve signaltonoise ratio.

We used a standard peak-detection approach and the general linear model implemented in SPM5 for our statistical analyses in order to identify the dynamic cerebral changes associated with cigarette craving, using a block design. First, we undertook a first-level analysis for each participant to investigate individual brain activation maps associated with our contrast of interest (appetitive smoking-related versus neutral material), and an autoregressive model (AR1) was implemented to account for serial correlations. A second-level random-effects model was then implemented to investigate the pattern of activations during the craving contrast in our 2 groups (females and males), using a one-sample *t*-test. Between-group differences were investigated using two-sample *t*-tests. Differences between the follicular and luteal phases were assessed using paired *t*-tests. As this is a pilot study, we set the threshold level for statistical significance at *P* < 0.001 (uncorrected) and extent thresholds of 10 contiguous voxels. We also reported results corrected for multiple comparisons using a Monte Carlo simulation computed with AFNIs 3dClustSim [23]. Assuming a per voxel probability threshold of *P* = 0.001, after 10000 simulations, a cluster size of 23 contiguous resampled voxels was indicated to correct for multiple comparisons at *P* < 0.05. Finally, using the Volume of Interest tool in SPM, we extracted the first eigenvariate from the one-sample *t*-test based on the center of each cluster identified by SPM. We then performed Pearson correlation analyses with the* Statistical Package for the Social Sciences* (SPSS) to investigate the association between hormonal levels and first eigenvariates of each region found to be significantly activated.

### 2.5. Behavioral Data Analyses

Potential differences between male and female smokers in sociodemographic data, basal and cue-induced cravings, and anxiety and depressive symptoms were assessed using independent *t*-tests. Differences in cue-elicited cravings between the follicular and luteal phases were examined using a paired *t*-test. We also performed Pearson correlation analyses between hormonal levels and ratings of craving with SPSS. (Note: for both fMRI and behavioral data, correlation analyses with STAI and BDI scores were not performed, since female and male smokers equally reported low levels of anxiety and depressive symptoms.)

## 3. Results

### 3.1. Sociodemographic Data

Men (M) and women (W) did not differ in age (M: mean = 30.2 ± 9.8; W: 32.2 ± 8.7; *P* = 0.543), years of education (M: 12.3 ± 2.1; W: 13.4 ± 2.8; *P* = 0.244), cigarettes per day (M: 20.0 ± 5.1; W: 17.9 ± 6.0; *P* = 0.290), years of smoking (M: 14.3 ± 9.8; W: 15.9 ± 9.5; *P* = 0.633), or the number of quitting attempts (M: 2.8 ± 2.8; W: 2.7 ± 2.6; *P* = 0.915).

### 3.2. Self-Report Data

Men and women did not differ in depressive symptoms (BDI) (M: 5.7 ± 6.4; W: 5.7 ± 6.4; *P* = 0.997), anxiety (STAI) (M: 35.8 ± 5.7; W: 35.8 ± 9.4; *P* = 0.991), basal cravings (FTCQ) (M: 3.8 ± 1.1; W: 3.6 ± 1.0; *P* = 0.680), or cue-elicited cravings (M: 48.3 ± 29.5; W: 41.6 ± 28.6; *P* = 0.505). However, there was a trend towards increased tobacco dependence severity in males, compared to females (FTND) (M: 5.2 ± 2.2; W: 3.5 ± 2.5; *P* = 0.056). There were no significant differences in ratings of cue-elicited cravings between the follicular (46.55 ± 31.45) and luteal phases (40.09 ± 29.09), in female participants. Finally, hormonal levels did not correlate with basal and cue-elicited cravings, within groups and menstrual phases.

### 3.3. Hormonal Levels

Hormonal levels were the following: estradiol (M: 96 pmol/L ± 43; W-follicular: 161 pmol/L ± 62; W-luteal 420 pmol/L ± 354), progesterone (M: 1.2 nmol/L ± 0.4; W-follicular: 1.6 nmol/L ± 2.1; W-luteal 12.5 nmol/L ± 15.4), and testosterone (M: 19.2 nmol/L ± 5.8; W-follicular: 2.6 nmol/L ± 0.8; W-luteal 1.8 ngmol/L ± 0.7).

### 3.4. fMRI Data: Sex Differences

*Men and Women One-Sample t* *-Tests.* As illustrated in Table 1, in females (during their first scanning session regardless of their menstrual cycle), cue-induced cravings elicited significant loci of activations in the left anterior cingulate gyrus (ACC) and precuneus. In males, significant activations were observed in the left (medial) superior frontal gyrus, ACC, insula, and putamen. Finally, hormonal levels did not correlate with craving-related brain activations, within groups.

*Two-Sample t* *-Test*. No increased activations were observed in females, relative to males, or vice-versa.

### 3.5. fMRI Data: Menstrual Phase in Females

**Figure 1:**
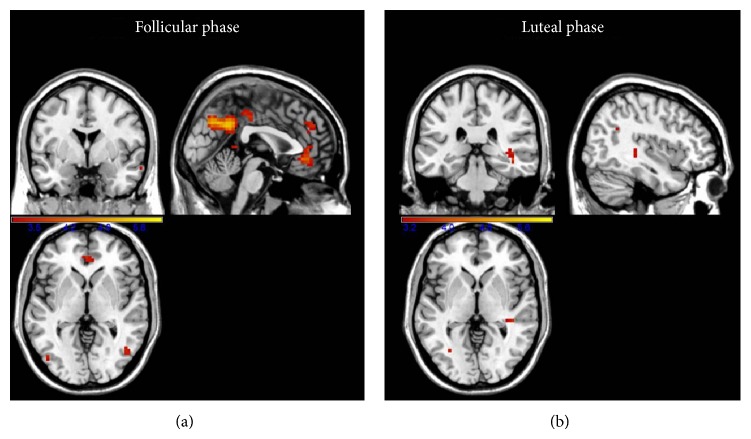
Cerebral activations of female participants, during viewing of appetitive cigarette images (relative to neutral images) and during the follicular and luteal menstrual cycle phases.

*One-Sample t* *-Tests.* During the follicular phase, female participants had significant activations in the bilateral angular gyrus, the left posterior cingulated gyrus, the right anterior cingulate gyrus, the right inferior temporal gyrus, the left precuneus, the left medial superior frontal gyrus, and the left middle cingulate gyrus. In contrast, during the luteal phase, the passive viewing of smoking cues only recruited significant activations in the right hippocampus (Table 2, Figure 1). Finally, hormonal levels did not correlate with craving-related brain activations, within menstrual phases.

*Paired t* *-Test*. In females, we found increased loci of activations in the right angular gyrus/middle temporal gyrus (*x* = 46; *y* = −63; *z* = 21; *Z*-score = 3.58; 22 voxels) and the right insula (*x* = 42; *y* = −14;  *z* = −4; *Z*-score = 3.11; 10 voxels) in the follicular phase, compared to the luteal phase. None of these regions were deactivated during the luteal phase (neutral minus cravings contrast). Finally, no increased activations were found in the luteal phase, relative to the follicular phase.

## 4. Discussion

In the present pilot study we examined sex/gender differences in the functional neuroanatomy of craving for cigarettes in smokers, as well as the fluctuations in craving-associated neural activations across the menstrual cycle in women. Analysis of the fMRI and self-reported craving data did not reveal any significant sex differences in the cerebral activations associated with craving or in the subjective ratings of the smoking-related stimuli. Nevertheless, the pattern of brain activations in women varied across their menstrual cycle, with extensive activations during the follicular phase (including cingulate, temporal gyrus, angular gyrus, precuneus, and medial superior frontal gyrus) and restricted activations during the luteal phase (only right hippocampus). Finally, the comparison between menstrual phases showed that there were increased activations in the angular gyrus in the follicular relative to the luteal phase.

The findings of greater cerebral reactivity to cigarette-related cues during the follicular relative to the luteal phase in women are in line with reports of fluctuations in tobacco intake and withdrawal symptoms, as well as relapse rates across the menstrual cycle in women [1, 13, 24–27]. However, the results of existing reports have been variable. Not all the studies have found the effect [12, 28], and a minority of studies have actually shown the reverse (e.g., increased cravings during the luteal phase) [29]. The preclinical studies of drug abuse have been more reliable, showing that higher levels of estrogen are associated with more reinforcing effects of addictive drugs, whereas higher levels of progesterone are associated with less reinforcing effects [30]. While the greater cerebral reactivity of female smokers in the follicular relative to the luteal phase of the menstrual cycles is somewhat unsurprising, the reasons explaining why the main differences were observed in the right angular gyrus remain elusive. Found to be activated in several fMRI studies on tobacco craving, the angular gyrus may contribute to the allocation of attention to salient stimuli and/or the evaluation of the self-relevance of cigarettes cues [9, 31]. Interestingly, recent resting-state studies in nicotine dependent and nonsmokers have shown both sex and menstrual cycle phase differences in the functional coupling of the inferior parietal cortex (angular gyrus) with other regions known to be implicated during introspective and self-referential thoughts [32, 33].

In the present study, we did assess hormonal levels and we did correlate them with the subjective cravings and with the neuroimaging data, but we failed to observe positive associations. However, the number of tested participants might have been too small to detect any significant relationships. Alternatively, in human smokers, the hormonal influences and biological forces overall may be much less important than psychological and sociocultural factors implicated in drug use and addiction.

It should be emphasized that the lack of differences between men and women in craving for cigarettes in the present study may be due to the fact that the participants were tested in satiety, while most robust sex differences in cue-induced craving have been found following abstinence for more than 12 hours [34, 35]. One of the reasons that may explain why sex differences in cue-induced cravings are more consistently found in studies involving abstinent versus satiated smokers is the presence of sex differences in withdrawal-related depressive symptoms. Consistently with this view, Allen et al. recently showed, in 147 abstinent smokers tested during the follicular and luteal phases, that depressive symptoms were more strongly associated with smoking urges than menstrual phase* per se* [36].

The current study had three main limitations. First, the study comprised a relatively small sample size, which limits the generalizability of our results, which may explain why we failed to observe between-group differences and why we only observed subtle differences in brain activations between the follicular and luteal phases. Another limitation of the study was that it involved smokers who were not abstinent, which may have decreased our chances of finding differences between male and female smokers. Finally, as this was a pilot study, we used a liberal statistical threshold, which increases the risk of committing type-I errors. However, we also applied a cluster size correction and most of our fMRI results remained significant.

## 5. Conclusions

The main finding of the present pilot study suggests that brain function associated with craving for cigarettes fluctuates across the menstrual cycle in women smokers. This result emphasizes the need for gender-specific programs to quit smoking, as well as taking into consideration a menstrual cycle phase during addiction treatment in women. More studies are needed to investigate biological and psychosocial factors that contribute to sex/gender differences in tobacco smoking.

## Figures and Tables

**Table 1:** Cerebral activations of male and female participants during viewing of craving images (relative to neutral images). R = right; L = left; BA = Brodmann area; *P* is uncorrected at 0.001.

Brain region	R/L	BA	MNI coordinates			*Z*-scores	Voxels^*^
			*x*	*y*	*z*		
Males							
Frontal superior medial	L	9	−4	52	14	3.48	233^**^
Anterior cingulate	L	9	−10	46	10	3.30	233^**^
Insula	L	—	−35	10	−7	3.38	30
Frontal superior	L	9	−21	56	24	3.19	51
Putamen	L	—	−28	0	14	3.17	28
Females							
Anterior cingulate	L	24	0	38	−7	3.27	29
Precuneus	L	23	−7	−60	21	3.10	40

^*^All results remained significant after application of cluster threshold as determined by Monte Carlo simulation; ^**^part of the same cluster.

**Table 2:** Cerebral activations of female participants, during viewing of appetitive cigarette images (relative to neutral images) and during the follicular and luteal menstrual cycle phases. R = right; L = left; BA = Brodmann area; *P* value is uncorrected at 0.001.

Brain region	R/L	BA	MNI coordinates			*Z*-scores	Voxels
			*x*	*y*	*z*		
Follicular phase							
Angular	R	39	49	−66	32	4.62	87
Posterior cingulate	L	31	−4	−49	32	3.98	241
Anterior cingulate	R	24	4	35	−7	3.45	77
Inferior temporal	R	19	46	−70	−4	3.40	21^*^
Precuneus	L	29	−7	−49	10	3.29	22^*^
Angular	L	39	−49	−66	38	3.21	50
Medial superior frontal	L	8	−4	46	32	3.20	33
Midcingulum	L	31	0	−28	42	3.04	21^*^
Luteal phase							
Hippocampus	R	—	42	−35	−10	3.36	15^*^

^*^These results are no longer significant after applying the cluster threshold as determined by Monte Carlo simulation.

## References

[1] Allen A. M., Allen S. S., Lunos S., Pomerleau C. S. (2010). Severity of withdrawal symptomatology in follicular versus luteal quitters: the combined effects of menstrual phase and withdrawal on smoking cessation outcome. *Addictive Behaviors*.

[2] Lombardi E. M. S., Prado G. F., de Paula Santos U., Fernandes F. L. A. (2011). Women and smoking: risks, impacts, and challenges. *Jornal Brasileiro de Pneumologia*.

[3] World Health Organization (2010). *Gender, Women, and the Tobacco Epidemic*.

[4] Institut de la Statistique du Québec (2009). *Enquête québécoise sur le tabac, l'alcool, la drogue et le jeu chez les élèves du secondaire, 2008*.

[5] Cepeda-Benito A., Reynoso J. T., Erath S. (2004). Meta-analysis of the efficacy of nicotine replacement therapy for smoking cessation: differences between men and women. *Journal of Consulting and Clinical Psychology*.

[6] Pierce J. P., Gilpin E. (1996). How long will today's new adolescent smoker be addicted to cigarettes?. *American Journal of Public Health*.

[7] Scharf D., Shiftman S. (2004). Are there gender differences in smoking cessation, with and without bupropion? Pooled- and meta-analyses of clinical trials of Bupropion SR. *Addiction*.

[8] Field M., Duka T. (2004). Cue reactivity in smokers: the effects of perceived cigarette availability and gender. *Pharmacology Biochemistry and Behavior*.

[9] Engelmann J. M., Versace F., Robinson J. D., Minnix J. A., Lam C. Y., Cui Y., Brown V. L., Cinciripini P. M. (2012). Neural substrates of smoking cue reactivity: a meta-analysis of fMRI studies. *NeuroImage*.

[10] Knott V., Cosgrove M., Villeneuve C., Fisher D., Millar A., McIntosh J. (2008). EEG correlates of imagery-induced cigarette craving in male and female smokers. *Addictive Behaviors*.

[11] McClernon F. J., Kozink R. V., Rose J. E. (2008). Individual differences in nicotine dependence, withdrawal symptoms, and sex predict transient fMRI-BOLD responses to smoking cues. *Neuropsychopharmacology*.

[12] Allen S. S., Hatsukami D. K., Christianson D., Nelson D. (1999). Withdrawal and pre-menstrual symptomatology during the menstrual cycle in short-term smoking abstinence: effects of menstrual cycle on smoking abstinence. *Nicotine & Tobacco Research*.

[13] Allen A. M., Mooney M., Chakraborty R., Allen S. S. (2009). Circadian patterns of ad libitum smoking by menstrual phase. *Human Psychopharmacology*.

[14] Fagerstrom K. O., Schneider N. G. (1989). Measuring nicotine dependence: a review of the Fagerstrom Tolerance Questionnaire. *Journal of Behavioral Medicine*.

[15] Berlin I., Singleton E. G., Heishman S. J. (2010). Validity of the 12-item French version of the tobacco craving questionnaire in treatment-seeking smokers. *Nicotine & Tobacco Research*.

[16] Beck A. T., Steer R. A., Ball R., Ranieri W. F. (1996). Comparison of Beck depression inventories -IA and -II in psychiatric outpatients. *Journal of Personality Assessment*.

[17] Spielberger C. D., Gorssuch R. L., Lushene P. R., Vagg P. R., Jacobs G. A. (1983). *Manual for the State-Trait Anxiety Inventory*.

[18] Mendrek A., Lakis N., Jiménez J. (2011). Associations of sex steroid hormones with cerebral activations during mental rotation in men and women with schizophrenia. *Psychoneuroendocrinology*.

[19] Mendrek A., Bourque J., Dubé A., Lakis N., Champagne J. (2012). Emotion processing in women with schizophrenia is menstrual cycle phase and affective valence dependent: an fMRI study. *ISRN Psychiatry*.

[20] Gilbert D. G., Rabinovich N. E. (1999). *International Smoking Image Series (With Neutral Counter-Parts), Version 1.2*.

[21] Lang P. J., Bradley M. M., Cuthbert B. N. (1997). *International Affective Picture System (IAPS): Technical Manual and Affective Ratings*.

[22] Friston K. J., Holmes A. P., Worsley K. J., Poline J.-P., Frith C. D., Frackowiak R. S. J. (1994). Statistical parametric maps in functional imaging: a general linear approach. *Human Brain Mapping*.

[23] Ward B. (2000). *Deconvolution Analysis of FMRI Time Series Data*.

[24] DeBon M., Klesges R. C., Klesges L. M. (1995). Symptomatology across the menstrual cycle in smoking and nonsmoking women. *Addictive Behaviors*.

[25] Pomerleau C. S., Garcia A. W., Pomerleau O. F., Cameron O. G. (1992). The effects of menstrual phase and nicotine abstinence on nicotine intake and on biochemical and subjective measures in women smokers: a preliminary report. *Psychoneuroendocrinology*.

[26] Mello N. K., Mendelson J. H. (1997). Cocaine's effects on neuroendocrine systems: clinical and preclinical studies. *Pharmacology Biochemistry and Behavior*.

[27] Snively T. A., Ahijevych K. L., Bernhard L. A., Wewers M. E. (2000). Smoking behavior, dysphoric states and the menstrual cycle: results from single smoking sessions and the natural environment. *Psychoneuroendocrinology*.

[28] Pomerleau C. S., Teuscher F., Goeters S., Pomerleau O. F. (1994). Effects of nicotine abstinence and menstrual phase on task performance. *Addictive Behaviors*.

[29] Franklin T. R., Napier K., Ehrman R., Gariti P., O'Brien C. P., Childress A. R. (2004). Retrospective study: influence of menstrual cycle on cue-induced cigarette craving. *Nicotine and Tobacco Research*.

[30] Lynch W. J., Sofuoglu M. (2010). Role of progesterone in nicotine addiction: evidence from initiation to relapse. *Experimental and Clinical Psychopharmacology*.

[31] Moran-Santa Maria M. M., Hartwell K. J., Hanlon C. A., Canterberry M., Lematty T., Owens M., Brady K. T., George M. S. (2014). Right anterior insula connectivity is important for cue-induced craving in nicotine-dependent smokers. *Addiction Biology*.

[32] Wetherill R. R., Jagannathan K., Shin J., Franklin T. R. (2014). Sex differences in resting state neural networks of nicotine-dependent cigarette smokers. *Addictive Behaviors*.

[33] Petersen N., Kilpatrick L. A., Goharzad A., Cahill L. (2014). Oral contraceptive pill use and menstrual cycle phase are associated with altered resting state functional connectivity. *NeuroImage*.

[34] Leventhal A. M., Waters A. J., Boyd S., Moolchan E. T., Lerman C., Pickworth W. B. (2007). Gender differences in acute tobacco withdrawal: Effects on subjective, cognitive, and physiological measures. *Experimental and Clinical Psychopharmacology*.

[35] Xu J., Azizian A., Monterosso J. (2008). Gender effects on mood and cigarette craving during early abstinence and resumption of smoking. *Nicotine & Tobacco Research*.

[36] Allen S. S., Allen A. M., Tosun N., Lunos S., al'Absi M., Hatsukami D. (2014). Smoking- and menstrual-related symptomatology during short-term smoking abstinence by menstrual phase and depressive symptoms. *Addictive Behaviors*.

